# Half-life–extended recombinant coagulation factor IX–albumin fusion protein is recycled via the FcRn-mediated pathway

**DOI:** 10.1074/jbc.M117.817064

**Published:** 2018-03-09

**Authors:** Jenny Chia, Jade Louber, Isabelle Glauser, Shirley Taylor, Greg T. Bass, Steve K. Dower, Paul A. Gleeson, Anne M. Verhagen

**Affiliations:** From the ‡ CSL Limited, Research, Bio21 Molecular Science and Biotechnology Institute, Melbourne, Victoria 3010, Australia,; the § Department of Biochemistry and Molecular Biology, Bio21 Molecular Science and Biotechnology Institute, University of Melbourne, Melbourne, Victoria 3010, Australia, and; the ¶ Department of Biomedical Engineering, University of Melbourne, Melbourne, Victoria 3010, Australia

**Keywords:** albumin, coagulation factor, endocytosis, Fc receptor, intracellular trafficking, Hemophilia B, extended half-life, factor IX, neonatal Fc receptor (FcRn), recombinant coagulation factor IX albumin-fusion protein (rIX-FP)

## Abstract

The neonatal Fc receptor (FcRn) has a pivotal role in albumin and IgG homeostasis. Internalized IgG captured by FcRn under acidic endosomal conditions is recycled to the cell surface where exocytosis and a shift to neutral pH promote extracellular IgG release. Although a similar mechanism is proposed for FcRn-mediated albumin intracellular trafficking and recycling, this pathway is less well defined but is relevant to the development of therapeutics exploiting FcRn to extend the half-life of short-lived plasma proteins. Recently, a long-acting recombinant coagulation factor IX–albumin fusion protein (rIX-FP) has been approved for the management of hemophilia B. Fusion to albumin potentially enables internalized proteins to engage FcRn and escape lysosomal degradation. In this study, we present for the first time a detailed investigation of the FcRn-mediated recycling of albumin and the albumin fusion protein rIX-FP. We demonstrate that following internalization via FcRn at low pH, rIX-FP, like albumin, is detectable within the early endosome and rapidly (within 10–15 min) traffics into the Rab11+ recycling endosomes, from where it is exported from the cell. Similarly, rIX-FP and albumin taken up by fluid-phase endocytosis at physiological pH traffics into the Rab11+ recycling compartment in FcRn-positive cells but into the lysosomal compartment in FcRn-negative cells. As expected, recombinant factor IX (without albumin fusion) and an FcRn interaction–defective albumin variant localized to the lysosomal compartments of both FcRn-expressing and nonexpressing cells. These results indicate that FcRn-mediated recycling via the albumin moiety is a mechanism for the half-life extension of rIX-FP observed in clinical studies.

## Introduction

The neonatal Fc receptor (FcRn)^3^ is a major histocompatibility class I–like molecule that consists of a transmembrane heavy chain that is noncovalently associated with the common β2-microglobulin chain. It is expressed widely and has multiple and diverse functions. Initially identified for its role in maternal transfer of IgG, from circulation to the fetus and from ingested colostrum across the neonatal gut epithelium, it is now known to mediate IgG transcytosis across multiple membrane barriers and throughout the lifetime of a mammal (1, 2). Even more critical is the role of FcRn in the rescue of albumin and IgG from lysosomal degradation (3–6), resulting in a prolonged half-life of 19–21 days in humans and high plasma concentrations of 10 and 30 mg/ml, respectively (7, 8). In recent years, there has been significant interest in exploiting FcRn to extend the half-life of therapeutic proteins, with fusion to the Fc region of IgG or albumin providing an opportunity to engage with FcRn and escape lysosomal degradation (9–11).

Several studies have used fluorescence imaging to investigate FcRn-mediated recycling of IgG following either fluid-phase endocytosis or uptake via FcRn at acidic pH (12–16). The FcRn-IgG complex, supported by the acidic conditions of the early endosome, is sorted into common recycling endosomes that divert cargo away from lysosomes. Subsequent exocytosis and exposure to the neutral pH of the extracellular milieu allows IgG to dissociate from FcRn. Albumin and IgG interact with different residues within FcRn and can bind to FcRn concurrently (17). It is widely assumed that an equivalent pathway exists for FcRn-mediated salvage of albumin, although to date, there has been a lack of imaging studies that have directly demonstrated the intracellular transport and recycling of internalized albumin.

Importantly, there appear to be differences in the relative contribution of different cell types to FcRn-mediated IgG and albumin homeostasis. Whereas hematopoietic cells and endothelial cells account for the majority of FcRn-mediated IgG homeostasis and are likely to play some role in FcRn-mediated albumin homeostasis (18, 19), the kidney has also been identified as an important site for FcRn-mediated albumin homeostasis (20). Unlike IgG, significant amounts of the smaller protein albumin can pass through the podocyte slit diaphragm of the kidney into the glomerular filtrate. There, it is actively retrieved and internalized by the megalin–cubulin complex expressed on proximal tubular capillary cells and subsequently transcytosed by FcRn back into the circulation (21).

Hemophilia B is a congenital bleeding disorder caused by gene mutations within the X-linked gene encoding coagulation factor (F)IX and affecting ∼1:30,000 males. Until recently, the standard therapy for patients involves replacement therapy using recombinant FIX (rFIX) or plasma-derived FIX, with patients suffering from more severe symptoms requiring prophylactic administration of FIX concentrates to prevent spontaneous bleeding. However, as a result of the short serum half-life of traditional FIX treatments (∼18 h in humans) (22, 23), prophylactic regimes with these products required intravenous administration every 2–3 days (24).

To reduce the frequency of dosing, a recombinant fusion protein linking coagulation factor IX with albumin, rIX-FP, was developed and is now licensed for use in the United States, Canada, Europe, Australia, and Japan. rIX-FP is produced as a single protein with a cleavable linker between FIX and albumin. The short linker peptide, derived from an endogenous activation peptide in native FIX, enables *in vivo* cleavage of activated FIX from the albumin moiety by FXIa when required for coagulation (25, 26). rIX-FP has demonstrated prolonged pharmacokinetics and pharmacodynamics, when compared with rFIX in preclinical studies (25, 27, 28) and in clinical trials (29, 30). Most recently, a 4–5-fold half-life extension was demonstrated in phase III studies in patients with severe hemophilia B, translating to a once every 14 days dosing regime (31).

Previous biosensor analysis has shown that the albumin moiety of rIX-FP supports interaction with FcRn under acidic conditions.^4^ Furthermore, the half-life extension of rIX-FP *in vivo* recently observed in clinical trials is consistent with FcRn-mediated recycling. However, the proposed cellular mechanism of half-life extension has not been directly demonstrated. In this study, we have established *in vitro* cellular systems to investigate the interaction of rIX-FP (and other albumin- or Fc-fusion proteins) with FcRn and the recycling through the FcRn-mediated salvage system. Our results demonstrate that FcRn engages with rIX-FP at acidic pH, diverting it from the lysosomal degradation pathway into the recycling endosomes for transport out of the cell. These data provide strong support for the contribution of the FcRn salvage pathway to the prolonged half-life of the FIX–albumin fusion *in vivo* and provide a cell system to rapidly analyze a range of albumin fusion proteins for their recycling efficiency.

## Results

### rIX-FP binds to cell-surface–expressed FcRn in a pH-dependent manner, like IgG and albumin

**Figure 1.**
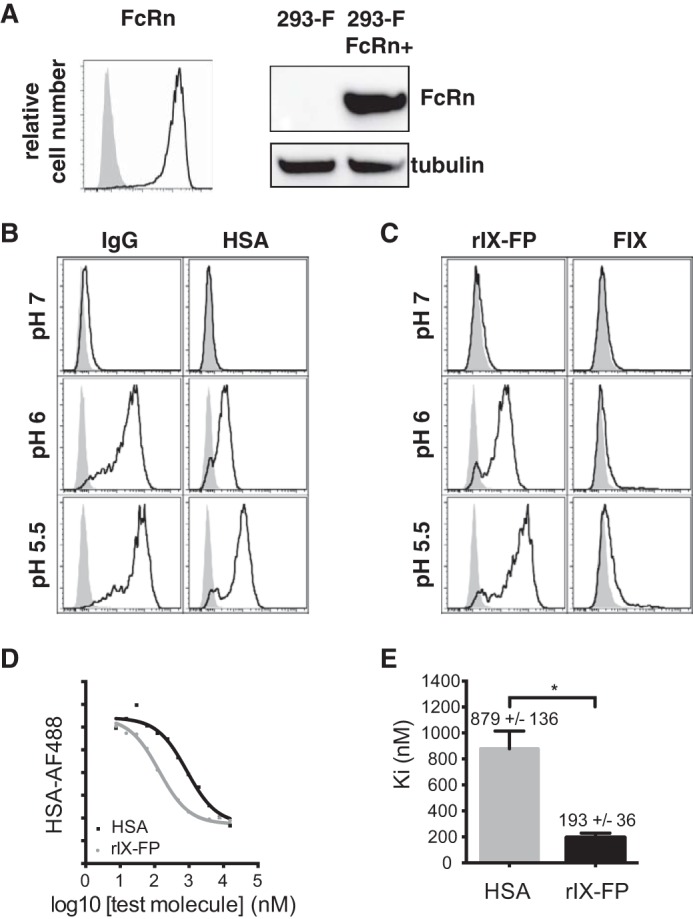
**rIX-FP binds to cell-surface–expressed FcRn in a pH-dependent manner, like IgG and albumin (HSA).** *A*, 293-F (*filled gray*) and 293-F FcRn+ (*black outline*) cells were analyzed by immunoblotting and flow cytometry analysis, using an anti-FcRn mAb. *B* and *C*, binding of cell-surface–expressed FcRn on 293-F FcRn+ cells (*black outline*) to fluorescently labeled IgG, albumin, rIX-FP, and rFIX was assessed at pH 5.5, 6, and 7.2. Binding to parental 293-F cells were included as negative controls (*filled gray*). *D*, relative binding of rIX-FP and albumin to cell-surface–expressed FcRn was assessed at pH 5.5 in competition-based inhibition assays, where 293-F FcRn+ cells were incubated with AF488-labeled albumin in the presence of increasing concentrations of unlabeled rHSA and rIX-FP. Data from a representative experiment are shown. *E*, relative binding of rIX-FP and albumin to FcRn, expressed as *K_i_* values (nm). The data represent the means ± S.E. from four independent competition-based inhibition experiments. *, *p* < 0.05

To investigate the interactions of albumin- and Fc-fusion proteins with FcRn, we generated a stable cell line expressing human FcRn and β2 microglobulin using FreeStyle^TM^ 293-F cells (henceforth, denoted by 293-F FcRn+). As shown in Fig. 1*A*, FcRn protein expression was validated by Western blotting and flow cytometric analysis. To verify the functionality of the cell-surface–expressed FcRn and the pH dependence of ligand engagement, IgG and albumin interaction with viable 293-F FcRn+ cells and the control parental line, 293-F, was examined over a pH range by flow cytometry. Specific binding of IgG and albumin to 293-F FcRn+ cells was minimal at neutral pH, but was readily detectable under mildly acidic conditions (pH 6), and was further enhanced at pH 5.5 (Fig. 1*B*); findings consistent with the pH-dependent interaction of ligands with FcRn.

Next, we compared the binding of rIX-FP and rFIX to cell-surface–expressed FcRn (Fig. 1*C*). Whereas specific interaction of rFIX to 293-F FcRn+ cells was not evident, binding of rIX-FP was detectable at pH 6 and further enhanced at pH 5.5. Hence, these results demonstrate that this albumin fusion, rIX-FP, has similar pH dependence for receptor binding to cells as unmodified albumin. Based on these results, pH 5.5 was selected for cell-based FcRn-binding studies.

To further examine the interaction of albumin and rIX-FP with cellular FcRn, we adapted a competition based inhibition assay from Mathur *et al.* (33), originally developed to evaluate the binding of IgG-based therapeutics for FcRn. In our assay, test molecules containing albumin compete with fluorescently labeled albumin (albumin-AF488) for binding to cell-surface–expressed FcRn at pH 5.5 (Fig. 1*D*), and the relative binding affinity to cell-surface–expressed FcRn is inferred by the individual *K_i_* values of the molecules. As shown in Fig. 1*E*, rIX-FP (*K_i_* of 193 ± 36 nm) binds to cell-surface–expressed FcRn with a stronger apparent affinity than albumin (*K_i_* of 879 ± 136 nm). Previous biosensor analyses using soluble FcRn have also derived a higher affinity for rIX-FP,^4^ although the difference between rIX-FP and albumin was only 2-fold (∼5 and 10 μm for rIX-FP and albumin, respectively, at pH 6). When examining ligand interaction with cell surface FcRn, however, it is possible that additional electrostatic or Gla domain–phospholipid interactions may occur, mediated by the FIX component of rIX-FP, thereby creating some binding avidity in the bifunctional fusion protein that may lower the *K_d_* (34). Nevertheless, these interactions are presumably too weak to be detectable for native FIX alone.

### Endogenous Rab11 is a marker for recycling endosomes and the FcRn-mediated recycling pathway in 293-F FcRn+ cells

**Figure 2.**
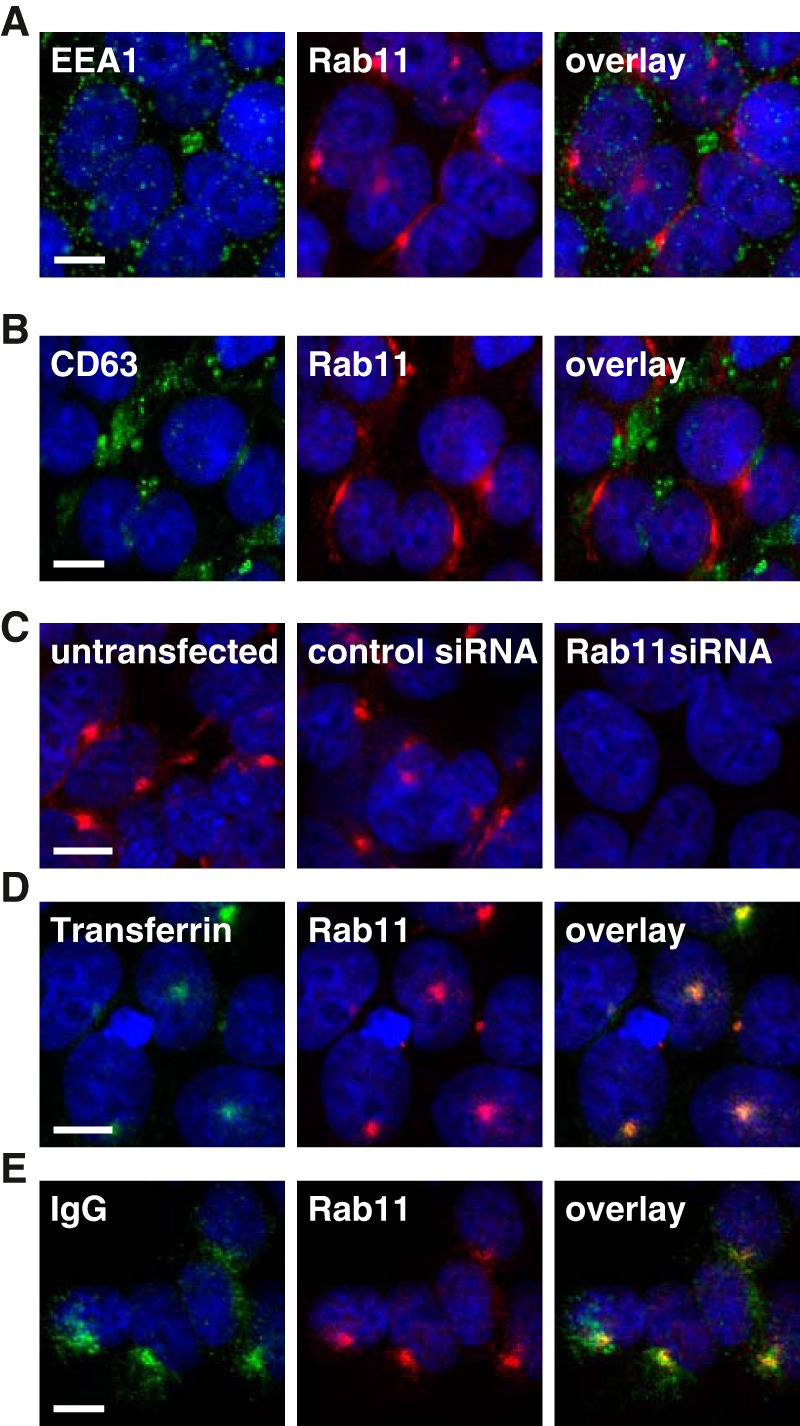
**Endogenous Rab11 is a marker for recycling endosomes and the FcRn-mediated recycling pathway in 293-F FcRn+ cells.** *A* and *B*, confocal images of 293-F FcRn+ cells stained with anti-EEA1 (*green*) and anti-Rab11 (*red*) antibodies (*A*) or anti-CD63 (*green*) and anti-Rab11 (*red*) antibodies (*B*). No co-localization was evident between the organelle-specific markers. *C*, scramble negative control and Rab11 siRNA transfected 293-F FcRn+ cells were stained with anti-Rab11 antibody. *D*, 293-F FcRn+ cells were pulsed with AF594-labeled transferrin (*green*) at neutral pH for 15 min at 37 °C, the cargo was removed, and the cells were incubated in complete media, pH 7, for a further 10 min, before fixing and staining with anti-Rab11 antibody (*red*). *E*, 293-F FcRn+ cells were pulsed with AF488-labeled IgG (*green*) at pH 5.5 for 10 min (37 °C), the cargo was removed, and the cells were incubated in complete media, pH 7, for a further 10 min, before fixing and staining with anti-Rab11 antibody (*red*). The cell nuclei were labeled with Hoechst 33342 (*blue*). All images were acquired on a Leica TCS SP5 confocal microscope with 63× magnification and a 1.4 NA oil-immersion objective as described in detail under “Experimental procedures.” Representative confocal middle sections are shown. *Scale bar*, 10 μm.

Having demonstrated the interaction between FcRn and the albumin/Fc-containing cargo on 293-F FcRn+ cells, we sought to determine whether receptor-bound cargo could then be internalized and recycled via the FcRn-mediated recycling pathway. To track the movement of internalized proteins through the intracellular recycling and/or degradation pathways in 293-F FcRn+ cells, we assessed a number of different antibodies raised against specific endosomal compartments that were suitable for paraformaldehyde-fixed samples. We selected a panel of three antibodies for co-localization experiments: anti-EEA1, anti-Rab11, and anti-CD63 as markers for the early endosomes, recycling endosomes, and late endosomes/lysosomes, respectively (Fig. 2, *A* and *B*). Importantly, co-staining cells with EEA1 (Fig. 2*A*) and CD63 (Fig. 2*B*) revealed minimal co-localization with Rab11.

Although EEA1 and CD63 antibodies have been used previously for identifying endosomal compartments in human cells, this is the first time, to the best of our knowledge, that a Rab11 antibody has been used to detect endogenous Rab11 in recycling endosomes as a marker for the FcRn-mediated recycling of cargo. Other publications have, however, used fluorescently tagged Rab11 constructs transfected into their cell line of interest (35). To confirm the specificity of the Rab11 antibody and validate its use in 293-F FcRn+ cells, we performed a knockdown experiment using siRNA against Rab11. Cells that were transfected with Rab11-specific siRNA resulted in almost complete loss of antigen detection by immunofluorescence (Fig. 2*C*), confirming antibody specificity.

To confirm whether 293-F FcRn+ cells are functionally capable of mediating intracellular trafficking and recycling, we examined IgG and transferrin, because the movement of these proteins via the Rab11 compartment through engagement with FcRn and transferrin receptors respectively is well documented. As shown in Fig. 2*D*, following internalization (10–15 min at pH 5.5 for IgG or pH 7 for transferrin) and a 10-min chase, both IgG and transferrin were shown to co-localize with Rab11+ recycling endosomes in 293-F FcRn+ cells, kinetics consistent with the trafficking of cargo from the cell surface to the recycling endosomes via the early endosomes.

### Like IgG, albumin is internalized and traffics via the early and recycling endosomes of the FcRn-mediated recycling process in 293-F FcRn+ cells

**Figure 3.**
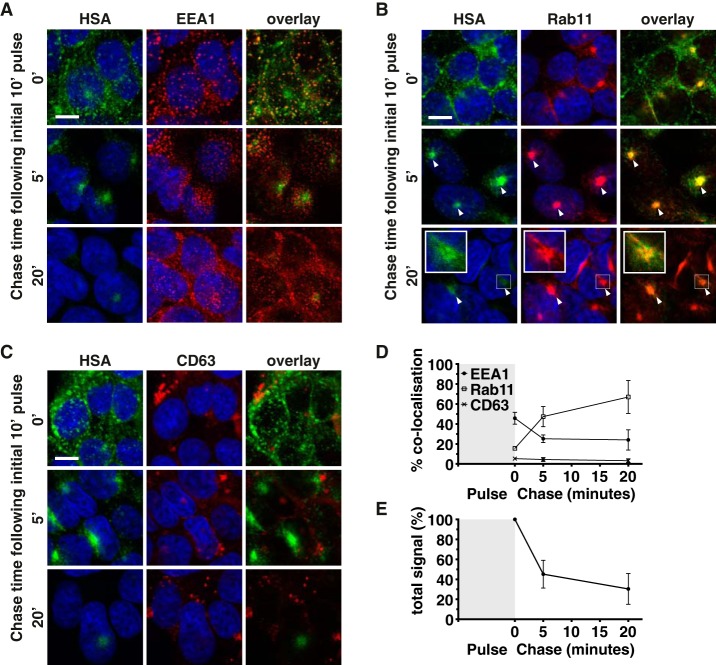
**Like IgG, albumin traffics via the early and recycling endosomes in 293-F FcRn+ cells.** *A–C*, 293-F FcRn+ cells were pulsed with AF488-labeled albumin at pH 5.5 (*green*) and chased for various times at pH 7. The cells were then fixed and stained with the following organelle specific markers (*red*): EEA1 for detection of early endosomes (*A*), Rab11 for recycling endosomes (*B*), or CD63 for late endosomes/lysosomes (*C*). The cell nuclei were labeled with Hoechst 33342 (*blue*). Representative confocal middle sections for each time point are shown. *Scale bar*, 10 μm. *Arrowheads* indicate co-localization. *Insets* show magnified images of selected areas denoted by the *white boxes*, where the signal for albumin has also been enhanced to highlight co-localization with Rab11. *D*, the proportion of albumin in each organelle after the 0-, 5-, and 20-min chase time points, as quantified according to “Experimental procedures” is expressed as % co-localization values. *E*, the amount of albumin remaining in 293-F FcRn+ cells at each time point is expressed as the relative number of objects/cell, as quantified according to “Experimental procedures.” For *D* and *E*, the data represent the means ± S.E. from three independent experiments, where for each experiment, an average value was determined from two to five images (each containing 13 or more cells) for each time point.

To evaluate the intracellular trafficking of albumin, 293-F FcRn+ cells were pulsed with AF488-labeled albumin at pH 5.5, for 10 min to allow the internalization of cargo via the surface FcRn receptor (minimal internalization by parental 293-F cells was observed under these conditions). Excess cargo was then removed and replaced with complete growth media (pH 7) for various chase periods. The cells were then fixed and stained for the organelle markers EEA1, Rab11, and CD63 (Fig. 3).

At the earliest time point (10-min pulse, 0-min chase), albumin was readily detectable within early endosomes (Fig. 3, *A*, *top row*, and *D*), decreasing thereafter. After a 5-min chase, the presence of cargo in the recycling endosome was clearly evident (Fig. 3, *B*, *second row*, and *D*). This was also observed at the 20-min chase period (Fig. 3, *B*, *third row*, and *D*), although the overall signal for albumin was markedly reduced, presumably because much had already been exported from the cells by this time (Fig. 3*E*). Co-localization at this later time point was observed as *orange* (rather than *yellow*/*pale green*) because of the dominance of the Rab11 signal (in *red*) over the faint albumin signal (in *green*) and is highlighted with *arrows* for individual and combined channels. By the 45-min chase, only traces of cargo were evident, consistent with observations for IgG (data not shown). Throughout the time course, minimal albumin was detected within the CD63+ lysosomal compartment (Fig. 3, *C* and *D*). Taken together, these data demonstrate that albumin is efficiently internalized and directed via the Rab11+ recycling endosomes in human cells expressing FcRn, similar to previous reports for IgG (12–16).

### rIX-FP also traffics via early and recycling endosomes in 293-F FcRn+ cells, with similar kinetics to albumin

**Figure 4.**
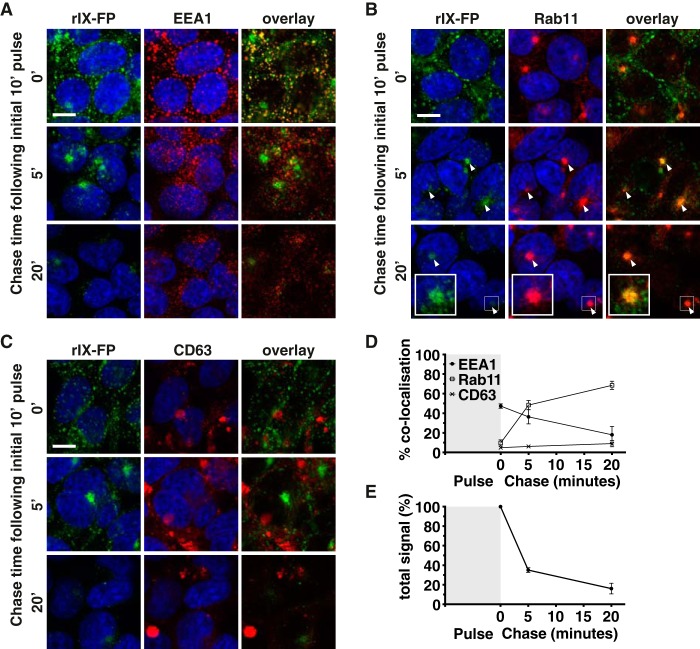
**rIX-FP traffics through 293-F FcRn+ cells via a similar route to albumin.** *A–C*, 293-F FcRn+ cells were pulsed with AF488-labeled rIX-FP at pH 5.5 (*green*) and chased for various times at pH 7. The cells were then fixed and stained with the following organelle specific markers (*red*): EEA1 for detection of early endosomes (*A*), Rab11 for recycling endosomes (*B*), or CD63 for late endosomes/lysosomes (*C*). The cell nuclei were labeled with Hoechst 33342 (*blue*). Representative confocal middle sections for each time point are shown. *Scale bar*, 10 μm. *Arrowheads* indicate co-localization. *Insets* show magnified images of selected areas denoted by the *white boxes*, where the signal for rIX-FP has also been enhanced to highlight co-localization with Rab11. *D*, the proportion of rIX-FP in each organelle after the 0-, 5-, and 20-min chase time points, as quantified according to “Experimental procedures,” is expressed as % co-localization values. *E*, the amount of rIX-FP remaining in 293-F FcRn+ cells at each time point is expressed as the relative number of objects/cell, as quantified according to “Experimental procedures.” For *D* and *E*, the data represent the means ± S.E. from three independent experiments, where for each experiment, an average value was determined from two to five images (each containing 13 or more cells) for each time point.

Next, 293-F FcRn+ cells were used to determine whether rIX-FP has the potential to be recycled via the FcRn pathway. As above for albumin, 293-F FcRn+ cells were pulsed with AF488-labeled rIX-FP at pH 5.5 for 10 min, and the transport of rIX-FP through intracellular organelles were examined after a series of chase intervals (Fig. 4). After the pulse (10-min pulse, 0-min chase), and as observed for albumin, rIX-FP was readily detected within the early endosome (Fig. 4, *A*, *top row*, and *D*). After a 5-min chase and from then on, rIX-FP was detected in the recycling endosome (Fig. 4, *B*, *second* and *third rows*, and *D*). As for albumin, after a 20-min chase, the amount of AF488-labeled rIX-FP cargo detected was markedly reduced (Fig. 4*E* and Fig. S1; *arrows* highlight regions of co-localization with Rab11+ endosome) and by 45 min was virtually undetectable (data not shown). Throughout the time course, rIX-FP was largely excluded from CD63+ lysosomal compartments (Fig. 4, *C* and *D*), suggesting minimal degradation.

**Figure 5.**
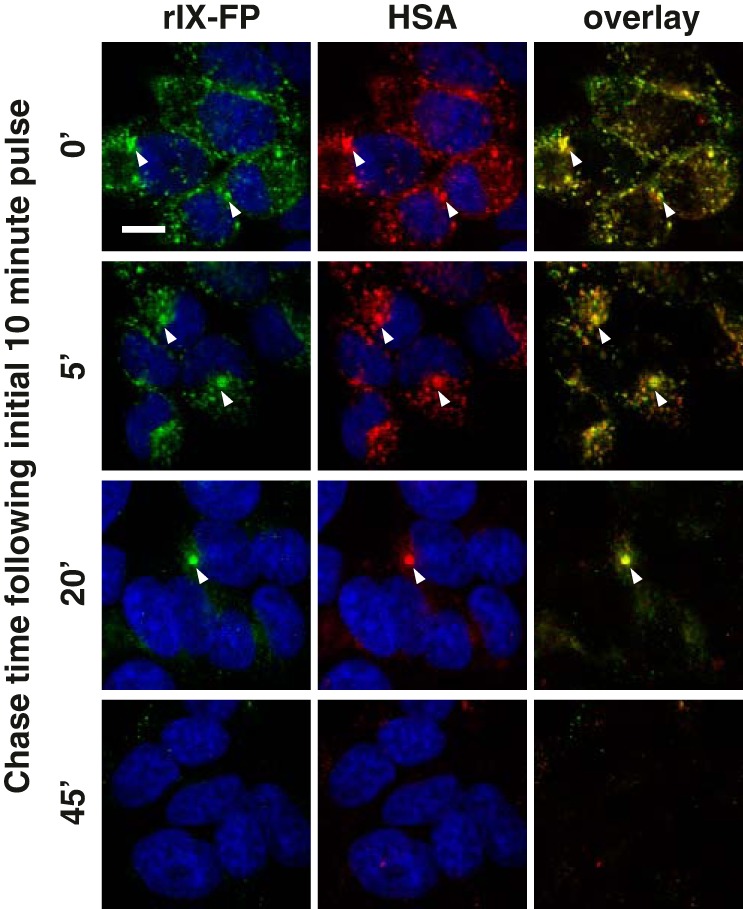
**Co-trafficking of HSA and rIX-FP in 293-F FcRn+ cells.** 293-F FcRn+ cells were pulsed simultaneously with AF488-labeled rIX-FP (*green*) and AF594-labeled albumin (*red*) at pH 5.5 for 10 min and chased in fresh complete media for various times at pH 7. The cells were then fixed, and the nuclei were stained with Hoechst 33342 (*blue*) and visualized by confocal microscopy. Representative confocal middle sections for each time point are shown, where results are representative of two independent experiments. *Scale bar*, 10 μm. *Arrowheads* indicate co-localization.

We also examined the co-trafficking of albumin and rIX-FP, where cells were simultaneously pulsed with differentially labeled cargoes at acidic pH (Fig. 5). Following FcRn-mediated uptake, the itinerary and kinetics of intracellular transport of AF594-labeled albumin and AF488-labeled rIX-FP were very similar, and a high degree of co-localization of the two molecules was observed.

### Albumin is rescued via FcRn interaction from lysosomal degradation following fluid-phase endocytosis, in 293-F FcRn+ cells

**Figure 6.**
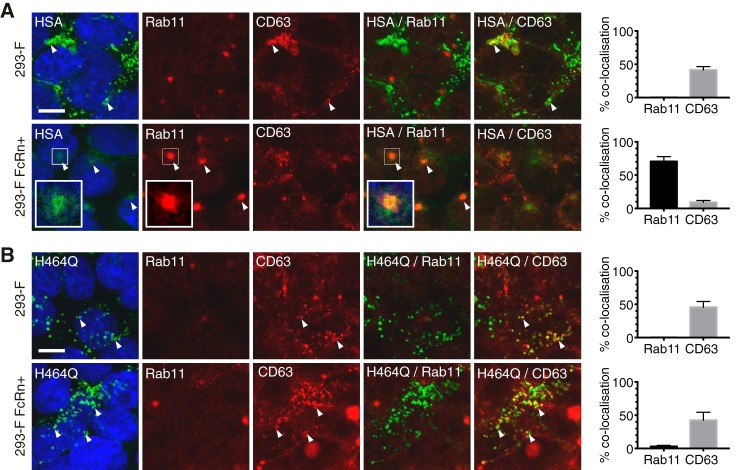
**Albumin is rescued from lysosomal degradation, following fluid-phase endocytosis, in cells expressing FcRn.** Fluid-phase uptake of albumin variants by 293-F and 293-F FcRn+ cells was examined. The cells were incubated with AF488-labeled albumin (HSA, *A*)) or FcRn interaction defective albumin variant H464Q (*B*) at neutral pH for 5–6 h, prior to fixation and staining with organelle markers (*red*). The cell nuclei were labeled with Hoechst 33342 (*blue*). Representative confocal middle sections are shown. *Scale bar*, 10 μm. *Arrowheads* indicate co-localization. *Insets* show magnified images of selected areas denoted by the *white boxes*, where the signal for albumin has also been enhanced to highlight co-localization with Rab11. The proportion of cargo in each organelle, as quantified according to “Experimental procedures,” is expressed as % co-localization values. The data represent the means ± S.E. from three independent experiments, where for each experiment, an average value was determined from two to five images (each containing 13 or more cells).

Although the internalization of albumin and IgG via FcRn at acidic pH may occur in some physiological settings, fluid-phase endocytosis, such as pinocytosis or macropinocytosis, is likely to have a greater role in internalization of these proteins *in vivo* from which subsequent rescue by FcRn may occur (36–38). To examine the cellular transport of albumin ligands under physiological conditions, cell monolayers were incubated with albumin at neutral pH for prolonged periods. Internalization of cargo was observed in both parental 293-F and 293-F FcRn+ cells, although the amount of cargo detected in the parental cells was notably higher (Fig. 6*A*; ∼3-fold increased levels of albumin in 293-F cells than 293-F FcRn+ cells). This is consistent with an absence of recycling in cells not expressing FcRn, where accumulation within the CD63+ compartments (where degradation occurs more slowly) was clearly evident (Fig. 6*A*, *top row*). In contrast, in 293-F FcRn+ cells, albumin was detected mainly in Rab11+ recycling endosomes with a weaker signal, presumably resulting from constant export from the cell and an absence of accumulation within the lysosomal compartment (Fig. 6*A*, *bottom row*). Co-localization with Rab11 was observed as *pale red*/*orange* given the dominance of the Rab11 signal (in *red*) over that of the ligand (*faint green*) (see *arrows* in Fig. 6*A*).

Under conditions of fluid-phase endocytosis, we were also able to investigate the intracellular fate of an albumin variant H464Q that cannot interact with FcRn (39). This mutant accumulated within the lysosomal compartment in both parental and 293-F FcRn+ cells and was not detected within the Rab11+ recycling endosome (Fig. 6*B*). Minimal difference was observed in the total signal detected between the two cells lines. These findings clearly demonstrate that the intracellular interaction of albumin with FcRn is essential for diversion of the endocytosed albumin from the endolysosomal pathway to the recycling endosomes.

### Following fluid-phase endocytosis, albumin fusion rescues rIX-FP from cellular degradation, in cells expressing FcRn

**Figure 7.**
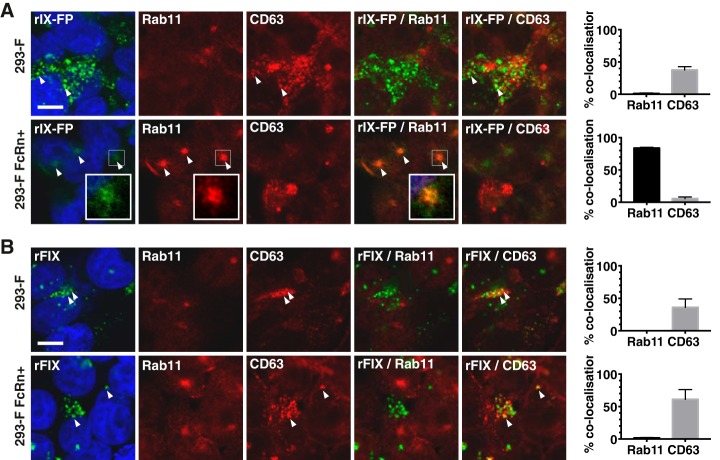
**FcRn dependent salvage of rIX-FP following internalization via fluid-phase endocytosis.** Fluid-phase uptake of FIX variants by 293-F and 293-F FcRn+ cells was examined. The cells were incubated with AF488-labeled rIX-FP (*A*) and rFIX (*B*) at neutral pH for 5–6 h, prior to fixation and staining with organelle markers (*red*). The cell nuclei were labeled with Hoechst 33342 (*blue*). Representative confocal middle sections are shown. *Scale bar*, 10 μm. *Arrowheads* indicate co-localization. *Insets* show magnified images of selected areas denoted by the *white boxes*, where the signal for rIX-FP has also been enhanced to highlight co-localization with Rab11. The proportion of cargo in each organelle as quantified according to “Experimental procedures” is expressed as % co-localization values. The data represent the means ± S.E. from three independent experiments, where for each experiment, an average value was determined from two to five images (each containing 13 or more cells).

Finally, we used our cellular system to determine the intracellular fate of rIX-FP following fluid-phase endocytosis. Like WT unmodified albumin, rIX-FP accumulated within the lysosome in the absence of FcRn expression and was detected within the Rab11+ compartment in 293-F FcRn+ cells (Fig. 7*A* and Fig. S2). The amount of cargo detected in the absence of exogenous FcRn expression was notably higher than in 293-F FcRn+ cells, again consistent with a lack of recycling in 293-F cells (∼3-fold more rIX-FP in 293-F cells). We also investigated the cellular fate of the unconjugated form of rFIX following fluid-phase endocytosis. Compellingly, rFIX accumulated within the lysosome in both parental and 293-F FcRn+ cells (Fig. 7*B*), and minimal difference was observed in the total signal detected between the two cell lines. Together, these results strongly support FcRn-mediated salvage via the albumin moiety as a mechanism for the half-life extension of rIX-FP observed in clinical studies.

## Discussion

The direct genetic fusion of biopharmaceuticals to albumin or the Fc portion of IgG has been increasingly explored in recent years as a strategy to improve plasma half-life. Several fusion proteins have been approved for use in various indications (9–11), including two albumin fusion proteins: Albiglutide, a GLP-1–albumin fusion protein for the treatment of type 2 diabetes (40), and rIX-FP for the treatment of hemophilia B. All have shown improved pharmacokinetics compared with nonfused counterparts in clinical studies, and although it is generally accepted that their half-life extension is due at least in part to FcRn-mediated salvage, FcRn recycling of albumin or albumin-fusion proteins at a cellular level has not been directly examined. In the current study, we have developed an *in vitro* cellular system that can be used to directly investigate the itinerary and kinetics of FcRn-mediated intracellular trafficking of albumin, IgG, and genetically engineered fusion proteins with albumin or Fc moieties.

Studies into the intracellular trafficking of cargo have commonly investigated recycling by co-localization with pathway-specific signature cargoes, such as transferrin and its receptor or, alternatively, the lack of co-localization with markers of the lysosomal degradation pathway (12, 13, 41–43). Recently, Schmidt *et al.* (43) have used this approach for investigations of albumin, demonstrating in cells overexpressing FcRn decreased co-localization with LAMP1+ lysosomes for a high binding albumin variant. In our study, we have investigated the recycling of albumin ligands and demonstrated its transit through the distinguishing organelle of this route, the recycling endosome (35, 44–46). The specificity of the Rab11 antibody used to directly detect endogenous Rab11, a Ras-like GTPase, as a marker for recycling endosomes was confirmed by the loss of signal in cells depleted of Rab11 using Rab11 siRNA, but not a control siRNA. In addition, following their uptake, transferrin and IgG were as previously reported found to move into the Rab11+ recycling compartment with rapid kinetics (Fig. 2).

Although multiple studies have demonstrated the itinerary for IgG recycling (12–14), this is the first time imaging studies have been thoroughly described for the recycling of albumin and fusion proteins thereof. Following internalization, both albumin and rIX-FP were shown to traffic via the early endosomes into the recycling endosomes before their disappearance from the cell, presumably as a consequence of cell-surface delivery and exocytosis. After a 20-min chase, signals for both albumin and rIX-FP were markedly reduced, and by 45 min, only traces remained. In addition, when cells were pulsed simultaneously with albumin and IgG, the two proteins followed the same intracellular trafficking route and with similar kinetics. Although the timing of endocytic routes has not been extensively described for FcRn recycling, the kinetics we observed are similar to those generally described for other recycling pathways (47).

Despite a 4–5-fold half-life extension, rIX-FP is cleared much more quickly than albumin (half-life of ∼102 h in humans (31) compared with 19 days for albumin (8)), suggesting a very active clearance mechanism for this coagulation factor. Although clearance pathways may be amenable to half-life extension via FcRn, recycling is a competitive process and less than 100% efficient. Therefore, a protein that undergoes rapid and continuous internalization will ultimately be more quickly degraded. The mechanism by which FIX is normally removed from the circulation is poorly understood. Although *in vitro* studies have suggested a role for the asialoglycoprotein receptor in binding and clearing FIX (48, 49), *in vivo* studies have demonstrated no difference in plasma levels of FIX in asialoglycoprotein receptor–deficient mice (49, 50). Activated FIX has been shown to interact with low-density lipoprotein–related protein-1 (LRP1) through an epitope exposed on the active protease, but an association has not been demonstrated for FIX zymogen (51), and no changes in FIX levels have been observed in mice deficient for LRP1 (52). The binding of FIX to vascular endothelial cells via collagen IV interaction has also been examined but does not appear to be the primary clearance pathway for FIX (53). A better understanding of the normal routes of clearance of FIX is clearly required to further elucidate the mechanisms and limitations of half-life extension of FIX through albumin fusion.

Although it remains a topic of debate (37, 38), it is generally accepted that fluid-phase endocytosis is the primary mediator of IgG and albumin entry into cells from plasma and would also be expected to have an important role in internalization of FIX as for any plasma protein. An essential role for endothelial and hematopoietic cells has been demonstrated in maintaining both IgG and albumin homeostasis using mice with tissue-specific Tie2 Cre-mediated deletion of FcRn (19). Because these cells are generally found in environments at near neutral pH when negligible binding of IgG and albumin to FcRn is observed, and given the very high levels of these proteins in plasma, the consensus view is that pinocytic uptake is the primary mediator of ligand entry into these cells. Albumin has also been shown to be able to be internalized by other cells, and the megalin–cubulin scavenger receptor, in particular, has been shown to have a critical role in salvage of albumin from glomerular filtrate by kidney tubular epithelial cells, followed by subsequent FcRn-mediated transcytosis back into the circulation (20, 54, 55). However, for cells bathed in acidic pH, such as neonatal gut epithelial cells, FcRn-mediated internalization of IgG and albumin may occur and is likely to mediate the transcytosis of maternal IgG to the neonatal circulation. Importantly, regardless of the uptake mechanism, an engagement of the ligand with FcRn within the acidic environment of the early endosomes, is essential for diverting the ligand away from the lysosomal degradation pathway and back to the cell surface, where exocytosis and a shift to neutral pH facilitates ligand release (13). Exocytosis of IgG has been shown to occur via different mechanisms, ranging from a complete fusion of exocytic vesicles with the plasma membrane (resulting in release all IgG at once), to a slower-release mode where secretory vesicles follow a complex form of kiss-and-run fusion (with only partial release of cargo at each event) (56). Consistently, we have demonstrated that albumin and rIX-FP, either internalized via FcRn at acidic pH or internalized by fluid-phase endocytosis, are directed through the intracellular recycling pathway in an FcRn-dependent manner (Figs. 4 and 7).

Importantly, albumin and Fc fusion technology can result in serum persistence in ways other than engaging the FcRn recycling machinery. An increase in the hydrodynamic volume of the therapeutic protein to prevent clearance through the kidney can play an important role, especially for small proteins. With a molecular mass of 55 kDa, it is possible that some FIX is normally cleared via kidney filtration, for which the cut-off is ∼70 kDa, but can also vary depending on the charge of the molecule. There is evidence for some clearance of coagulation factor VII, which is of a similar size and composition to FIX, through the kidney (57). It is therefore possible that prevention of clearance via the kidney may also contribute to the increased half-life of rIX-FP.

In conclusion, we present evidence to support the hypothesis that rIX-FP can exploit the FcRn-mediated recycling pathway normally reserved for IgG and albumin, providing a mechanism for the 4–5-fold half-life extension recently demonstrated in clinical trials (31). In addition, the cell-based assays we have described provide a valuable platform for assessing the recycling capacity of novel half-life–extended therapeutics.

## Experimental procedures

### Materials

Recombinant albumin (human) and monoclonal IgG (human IgG1) were expressed in FreeStyle^TM^ 293-F Cells (Life Technologies) and purified as previously described (58). rIX-FP (Idelvion) was obtained from CSL Behring (GmbH, Germany) and rFIX (BeneFIX®) from Pfizer Pharma (GmbH, Berlin, Germany). Human transferrin conjugated to Alexa Fluor® 594 (AF594) was purchased from Thermo Fisher (Molecular Probes, T13343).

Recombinant albumin, albumin variant H464Q, IgG, rIX-FP, and rFIX were labeled with Alexa Fluor® 488 (AF488) NHS ester (succinimidyl ester) (Life Technologies, A-20000) or Alexa Fluor® 594 (AF594) NHS ester (succinimidyl ester) (Life Technologies, A-37572), according to the manufacturer's protocol. For flow cytometry and Western blotting analysis, the following antibodies were used: mouse anti-FcRn antibody (Acris Antibodies, AM26754PU-N), anti-tubulin-HRP antibody (Abcam, ab185067), anti-mouse IgG-HRP (Jackson ImmunoResearch, 715-035-150), and anti-mouse IgG-AF488 (Molecular Probes, A-11029).

### Generation of stable FcRn-expressing cells

FreeStyle^TM^ 293-F cells were grown under adherent conditions in growth medium containing RPMI supplemented with GlutaMAX^TM^ (Gibco) and 10% fetal bovine serum (Sigma–Aldrich, 12003C) in a humidified 5% CO_2_ incubator at 37 °C. The cells were transfected with linearized plasmids containing human FcRn and β2 microglobulin sequences using Lipofectamine 2000 (Thermo Fisher, 11668019) and maintained in growth medium containing 0.5 mg/ml G418 (Thermo Fisher, 10131027). Single-cell clones were individually picked from transfection cultures and expanded under constant antibiotic selection. The clones with high expression levels were identified by on a FACS by binding to AF488-labeled human IgG at acidic pH. One of the high expressing clones was selected for use in binding, trafficking, and recycling assays (henceforth denoted by 293-F FcRn+).

To confirm FcRn expression, 293-F and 293-F FcRn+ cells were stained for 30 min on ice with 10 μg/ml mouse anti-FcRn antibody (Acris Antibodies, AM26754PU-N), followed by a 30-min incubation with secondary antibody, anti-mouse IgG-AF488 (4 μg/ml, Molecular Probes, A-11029). After the incubation, the cell-bound fluorescence was analyzed on a LSR Fortessa^TM^ cell analyzer (BD Biosciences). Expression was also confirmed by Western blotting analysis.

### Rab11 siRNA transient transfections

To knock down Rab11 in 293-F FcRn+ cells, siRNA duplexes targeting Rab11A (5′-AAUGUCAGACAGACGCGAAAA[dT][dT]-3′) and Rab11B (5′-AAGCACCUGACCUAUGAGAAC[dT][dT]-3′) (Sigma–Aldrich) were delivered to the cells at a final concentration of 50 nm, using DharmaFECT 1 transfection reagent (GE Dharmacon, T-2001-2). At 48 h post-transfection, the cells were analyzed for Rab11 expression by immunofluorescence microscopy, using a rabbit anti-Rab11 antibody (Abcam, ab3612). A nontargeting siRNA duplex (5′-AGGUCGGUGUGCUCUUGUUGG[dT][dT]-3′) (Sigma–Aldrich) was included as a negative control.

### In vitro cell-based FcRn-binding assays

For binding assays, 293-F FcRn+ cells were cultured in suspension in FreeStyle^TM^ 293 expression medium (Thermo Fisher, 12338018) supplemented with 0.1% Pluronic (Thermo Fisher, 24040032), antibiotic–antimycotic solution (Thermo Fisher, 15240), and 0.5 mg/ml G418 (Thermo Fisher, 10131027), in a humidified 8% CO_2_ orbital shaker–incubator (150 rpm), for at least 3–5 days prior to the experiment.

To assess the pH specificity of cargo binding to cell-surface–expressed FcRn, 293-F FcRn+ cells were resuspended in Dulbecco's PBS (Sigma–Aldrich, D8537) at a pH of 5.5, 6.0, or 7 (2 × 10^5^ cells/100 μl/reaction) and incubated with 50 nm of AF488-labeled material. After an hour of incubation on ice, the cells were washed in the same buffer of corresponding pH, before their cell-bound fluorescence was analyzed on a LSRFortessa^TM^ cell analyzer (BD Biosciences).

To perform the competition-based inhibition assay, 293-F FcRn+ cells were resuspended at a density of 8 × 10^6^ cells/ml in serum-free assay medium (Dulbecco's PBS at pH 5.5). The cells were then plated in U-bottom wells of a 96-well plate containing mixtures of labeled competitor molecule (albumin-AF488) and unlabeled test molecules (albumin or rIX-FP). In a typical assay, 25 μl of cell suspension (2 × 10^5^ cells/well), 25 μl of albumin-AF488 diluted from stock in assay media to give a final concentration of 1 μg/ml (15.15 nm), and 50 μl of varying concentrations of albumin-containing test molecule were added to wells in a total volume of 100 μl/well. The assay mixture was incubated for 2 h at 4 °C with constant shaking. After the incubation, the cell-bound fluorescence in each well was read on the LSR Fortessa^TM^ cell analyzer. The mean fluorescence intensity was obtained from each experiment and analyzed using GraphPad Prism. The equilibrium dissociation constant, *K_i_*, for each test molecule was determined according to the one-site fit *K_i_* model for competitive binding experiments.

### Intracellular trafficking assays following FcRn-mediated uptake at acidic pH

For intracellular trafficking assays, 293-F FcRn+ cells were plated in 8-well chamber Nunc^TM^ Lab-Tek^TM^II CC2^TM^ chamber slide system (Thermo Fisher, 154941) and grown to ∼80% confluency. Protein cargo was diluted in assay medium at the following concentrations: 20 μg/ml AF594-labeled transferrin, 0.1 μm AF488-labeled IgG, 2–4 μm AF488/AF594-labeled albumin, or 0.5–1 μm AF488-labeled rIX-FP and incubated with cells in a humidified 5% CO_2_ incubator at 37 °C. After a 10–15-min pulse at the specified pH (pH 5.5 for IgG, albumin, and rIX-FP; pH 7 for transferrin), the supernatant containing excess cargo (that had not been internalized) was removed and replaced with prewarmed complete growth medium. Internalized cargo was allowed to traffic for various time periods (chase) at 37 °C in a humidified 5% CO_2_ incubator, before incubation was stopped. The cells were fixed with 4% paraformaldehyde for 15 min and either visualized by confocal microscopy or further processed for intracellular staining of organelles.

For intracellular staining of organelles, the cells were permeabilized in 0.5% Triton X-100/PBS for 5 min and blocked in 1% BSA/PBS at 4 °C overnight. Monolayers were incubated with 0.5 μg/ml mouse anti-EEA1 (BD Biosciences, 610457), 0.2 μg/ml mouse anti-CD63 (Santa Cruz, sc-5275), or 2.5 μg/ml rabbit anti-Rab11 (Abcam, ab3612) for 1.5 h at room temperature or at 4 °C overnight, followed by a 45-min incubation with secondary antibodies, anti-mouse IgG-AF647 (4 μg/ml, Molecular Probes, A-21236), anti-rabbit IgG-AF647 (4 μg/ml, Molecular Probes, A-21245), and anti-rabbit IgG-AF546 (4 μg/ml, Molecular Probes, A-11035). After the intracellular staining (EEA1, Rab11, or CD63), the cells were incubated for 15 min with Hoechst 33342 diluted in PBS (10 μg/ml, Molecular Probes, H3570). Following the intracellular staining, the cells were mounted with ProLong Gold antifade mountant (Molecular Probes, P36930), and a coverslip was applied. The cells were examined using a Leica TCS SP5 confocal microscope (Leica Microsystems) equipped with DIC and fluorescence optics, diode 405-nm, argon 488-nm, diode pumped solid state 561-nm, and HeNe 633-nm lasers. The fluorescence images were collected with a 63× magnification and 1.4 NA oil-immersion objective at 37 °C using sPMT (spectral detectors) and T-PMT (transmitted light) and acquisition software LAS AF (Leica Application Suite Advanced Fluorescence) version 2.6.0.7266. Time-course images for each cargo were taken using the same laser intensity, exposure, and gain settings to allow for direct comparison.

Quantitation of the co-localization between internalized cargo and fluorescent organelle markers was performed using the plugin organelle-based co-localization as described by Woodcroft *et al.* (32), on the FIJI program (National Institutes of Health public domain software). Two to five images (each containing ≥13 cells) from each experiment were analyzed at every time point, because preliminary experiments revealed no significant difference in quantitation when cells were analyzed individually or together as an image. Images from each time-course experiment were analyzed under identical conditions, with constant threshold values used to identify the cargo and organelles. Co-localization values were expressed as a percentage of total cargo, determined by taking the sum of overlapping pixels between the cargo and respective markers, divided by the total number of cargo pixels. The average number of objects per cells was also calculated for each experiment by dividing the number of objects (defined as a minimum of five pixels) by the total number of cell nuclei per image and expressed relative to the first time point taken (T0). All data are expressed as means ± S.E. of three independent experiments.

### Fluid-phase endocytosis assays in 293-F and 293-F FcRn+ cells

293-F FcRn+ cells and 293-F cells were plated in 8-well chamber Nunc^TM^ Lab-Tek^TM^ II CC2^TM^ chamber slide system and grown to ∼80% confluency. Protein cargo was diluted in prewarmed complete growth medium at the following concentrations: 2 μm AF488-labeled albumin, 2 μm AF488-labeled albumin variant H464Q (39), 1 μm AF488-labeled rIX-FP, or 1 μm AF488-labeled rFIX and incubated with cells in a humidified 5% CO_2_ incubator at 37 °C. After a continuous pulse of 5–6 h, the supernatant containing excess cargo was removed, and cells were fixed and further processed for intracellular staining as described above. Imaging and quantitation of the co-localization between internalized cargo and fluorescent organelle markers was also performed as described above. Importantly, the laser intensity, exposure, gain, and threshold settings were kept constant for each cargo analyzed, to allow for direct comparison between cell lines.

## Author contributions

J. C. designed and performed research, analyzed and interpreted data, and wrote the manuscript. J. L. and I. G. designed and/or performed experiments and analyzed and interpreted data. S. T. contributed vital reagents. G. T. B. helped analyze quantitative data. S. K. D. and P. A. G. helped design research and edited the manuscript. A. M. V. designed research, analyzed and interpreted data, and wrote the manuscript.

## Supplementary Material

Supporting Information

## Footnotes

4 H. Braley, V. Tomasetig, S. Zollner, G. Dickneite, S. Schulte, S. Dower, and D. Hartman, poster presented at the World Federation of Hemophilia (WFH) World Congress, Paris, France (July 8–12, 2012).

## Footnotes

3 The abbreviations used are:

FcRn: neonatal Fc receptor
rIX-FP: recombinant coagulation factor IX–albumin fusion protein
FIX: coagulation factor IX
rFIX: recombinant FIX
LRP1: low-density lipoprotein–related protein-1
HSA: human serum albumin
AF: Alexa Fluor.
