# Supplementary material for: Half-life–extended recombinant coagulation factor IX–albumin fusion protein is recycled via the FcRn-mediated pathway
Source: J Biol Chem. 2018 Mar 9;293(17):6363–73. doi: 10.1074/jbc.M117.817064 (PMC5925791; doi:10.1074/jbc.M117.817064)
Supplement: Supporting Information [file supp_293_17_6363__index.html]

Half-life extended recombinant coagulation factor IX albumin fusion protein is recycled via the FcRn-mediated pathway — Half-life–extended recombinant coagulation factor IX–albumin fusion protein is recycled via the FcRn-mediated pathway — Half-life–extended rIX-FP is recycled via FcRn — Supporting Information 

# Half-life–extended recombinant coagulation factor IX–albumin fusion protein is recycled via the FcRn-mediated pathway

## Supporting Information

- Supplemental data (.pdf, 1.2 MB) - Supplemental data, including supplemental figure 1 and supplemental figure 2
