# Supplementary material for: Half-life–extended recombinant coagulation factor IX–albumin fusion protein is recycled via the FcRn-mediated pathway
Source: J Biol Chem. 2018 Mar 9;293(17):6363–73. doi: 10.1074/jbc.M117.817064 (PMC5925791; doi:10.1074/jbc.M117.817064)
Supplement: Supporting Information [file 10.1074_M117.817064_jbc.M117.817064-1.pdf]

Supplementary data

Half-life extended recombinant coagulation factor IX albumin fusion protein is recycled via the FcRn-mediated pathway

Jenny Chia[1], Jade Louber[2#], Isabelle Glauser[1#], Shirley Taylor[1], Greg T Bass[1,3], Steve K Dower[1], Paul A Gleeson[2], and Anne M Verhagen[1*]

From [1]CSL Limited, Research, Bio21 Molecular Science and Biotechnology Institute, Melbourne, Australia; [2]Department of Biochemistry and Molecular Biology, Bio21 Molecular Science and Biotechnology Institute, University of Melbourne, Melbourne, Australia; [3]Department of Biomedical Engineering, University of Melbourne, Melbourne, Australia

[#]Authors contributed equally to this study

List of materials:

Supplementary Figure 1
Supplementary Figure 2

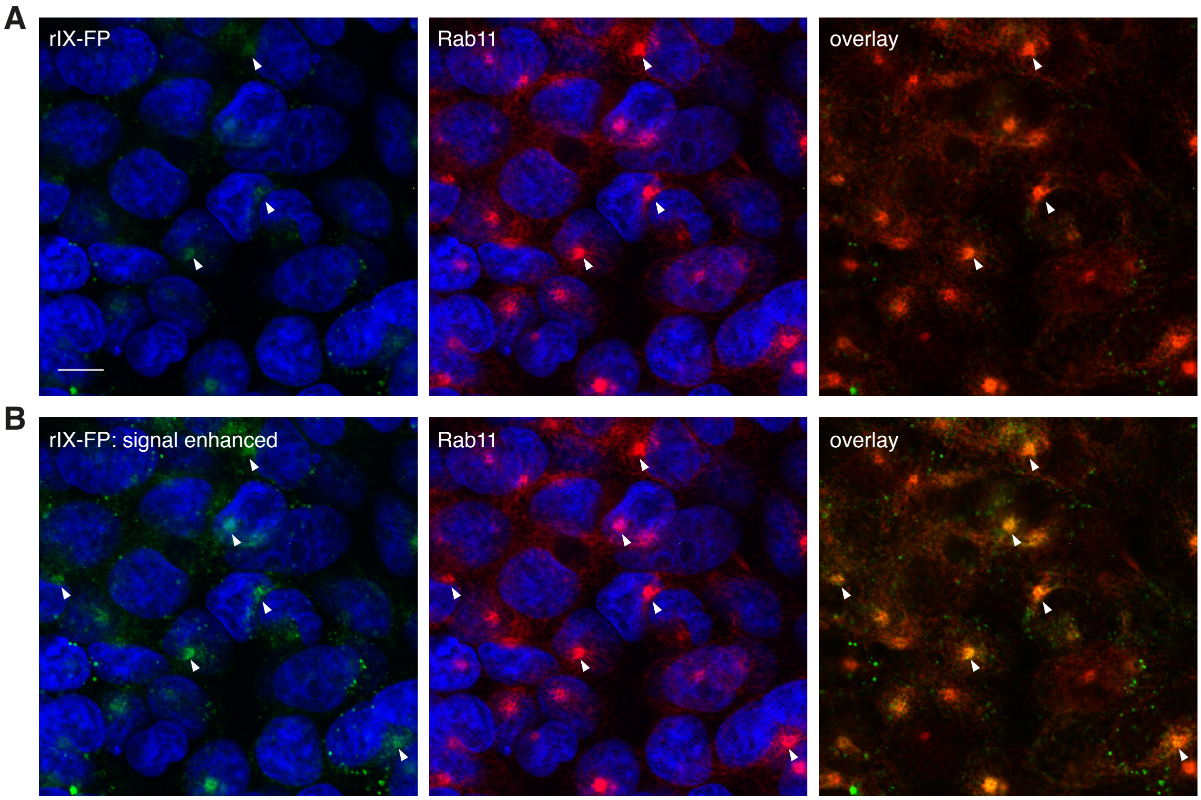

Supplemental Figure 1. rIX-FP traffics through Rab11+ recycling endosomes, following internalization at pH 5.5. 293-F FcRn+ cells were pulsed with AF488-labelled rIX-FP at pH 5.5 (green) and chased for 20 minutes at pH 7. Cells were then fixed and stained with Rab11 (red) for recycling endosomes, and with Hoechst 33342 (blue) for cell nuclei. (A) By 20 minutes, the majority of rIX-FP has left the cell and remaining signal is quite weak. Nevertheless, a high degree of co-localisation with Rab11 is evident, as indicated with arrowheads and further depicted in (B), where the the signal for rIX-FP has been enhanced. Representative confocal middle sections are shown. Scale bar, 10 µm. Arrowheads indicate co-localization.

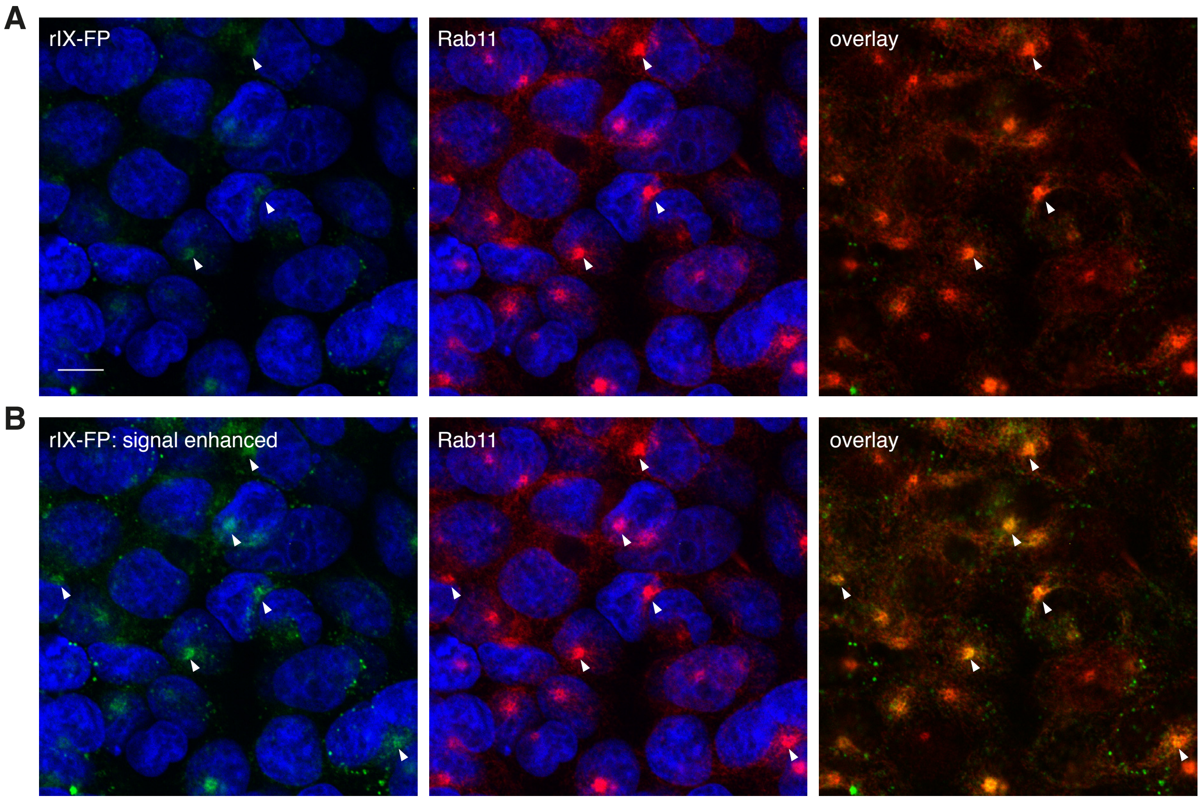

Supplemental Figure 2. rIX-FP is recycled via the FcRn-mediated pathway, following internalization via fluid phase endocytosis. 293-F FcRn+ cells were incubated with AF488-labelled rIX-FP at neutral pH for 5-6 hours, prior to fixation and staining with Rab11 antibodies (red) for recycling endosomes, and with Hoechst 33342 (blue) for cell nuclei. (A) The signal for rIX-FP is less strong in 293-F FcRn+ cells compared to the parental cells (refer to Figure 7), as the protein is continually recycled out of the cells via FcRn. Nevertheless, a high degree of co-localisation is clearly evident as indicated with arrowheads and further depicted in (B), where the the signal for rIX-FP has been enhanced. Representative confocal middle sections are shown. Scale bar, 10 µm. Arrowheads indicate co-localization.
